# Older Age Is Associated with Decreased Levels of VDR, CYP27B1, and CYP24A1 and Increased Levels of PTH in Human Parathyroid Glands

**DOI:** 10.1155/2020/7257913

**Published:** 2020-04-09

**Authors:** Yi Jiang, Liyan Liao, Jina Li, Larry Wang, Zhongjian Xie

**Affiliations:** ^1^Department of Pathology, The Second Xiangya Hospital, Central South University, Changsha, Hunan, China; ^2^Department of Thoracic Surgery, The Second Xiangya Hospital, Central South University, Changsha, Hunan, China; ^3^Department of Pathology, Children's Hospital Los Angeles, University of Southern California, Los Angeles, CA 90027, USA; ^4^Department of Metabolism and Endocrinology, Hunan Provincial Key Laboratory of Metabolic Bone Diseases, National Clinical Research Center for Metabolic Diseases, The Second Xiangya Hospital of Central South University, 139 Middle Renmin Road, Changsha 410011, Hunan, China

## Abstract

Parathyroid glands contain the vitamin D receptor (VDR) and 25-hydroxyvitamin D-1*α*-hydroxylase (CYP27B1) and 24-hydroxylase (CYP24A1), which catalyze the production and degradation of 1,25-dihydroxyvitamin D [1,25(OH)_2_D], respectively. Previous studies have shown that the serum level of intact parathyroid hormone (iPTH) increases with age. We hypothesized that the expression of CYP27B1 or VDR in parathyroid glands decreases with age, which might account for the increased serum levels of iPTH due to decreased suppression of parathyroid hormone (PTH) secretion by 1,25(OH)_2_D in older people. To test this hypothesis, we examined relative expression levels of VDR, CYP27B1, CYP24A1, and PTH in specimens from parathyroid glands unintentionally removed during thyroidectomy for 70 patients varying in age from 10 to 70 years. The results showed that there was an inverse correlation between age and VDR, CYP27B1, and CYP24A1 expression (*p* < 0.05). A significant positive correlation between PTH expression levels and age was also observed (*p* < 0.05). These data indicate that older age is associated with decreased levels of VDR, CYP27B1, and CYP24A1 and increased levels of PTH in human parathyroid glands.

## 1. Introduction

Vitamin D is hydroxylated by 25-hydroxylase in the liver to 25-hydroxyvitamin D [25(OH)D] that is subsequently hydroxylated by 25-hydroxyvitamin D-1*α*-hydroxylase (CYP27B1) in the kidney to 1,25-dihydroxyvitamin D [1,25(OH)_2_D]. The latter is the active hormone that plays an important role in maintaining blood calcium and phosphorus levels and skeletal mineralization. 1,25(OH)_2_D is catabolized to 1,24,25(OH)_3_D by vitamin D-24-hydroxylase (CYP24A1) in target cells. It has been shown that 1,25(OH)_2_D inhibits the synthesis and secretion of parathyroid hormone (PTH) and prevents proliferation of parathyroid glands [1, 2]. Serum levels of intact parathyroid hormone (iPTH) in the elderly population are reported to be higher than those in the younger population [3–5].

Parathyroid glands contain the vitamin D receptor (VDR) [6, 7], CYP27B1, and CYP24A1 [8]. Previous studies have shown that the expression of VDR decreases with age in cultured skeletal myocytes [9], human muscle tissue [10], rat intestine [11, 12], bone [11], and mammary glands [13]. However, it is unknown whether the expression of VDR, CYP27B1, or CYP24A1 is altered in parathyroid glands in older people. To address this question, we examined the expression levels, VDR, CYP27B1, CYP24A1, and PTH in human parathyroid glands in 70 patients undergoing thyroidectomy varying in age from 10 to 70 years.

## 2. Subjects and Methods

### 2.1. Parathyroid Specimens

Parathyroid specimens were collected from 70 parathyroid glands stored in the Department of Pathology of the Second Xiangya Hospital of Central South University. These 70 parathyroid glands were unintentionally removed during 6545 thyroidectomies performed between 2012 and 2016. Patients who underwent thyroidectomy had a diagnosis of the nodular goiter as a primary reason for surgery. All operations were performed at the Department of General Surgery, the Second Xiangya Hospital of Central South University. The study was approved by the Ethics Committee of the Second Xiangya Hospital of Central South University. Clinical records for all patients collected are detailed in Table 1. Sixteen specimens were from male patients, and 54 specimens were from female patients. The mean age was 41 ± 14 years. The serum calcium, phosphate, hepatorenal function, and intact PTH level were measured as the routine preoperative examination. All patients had normal levels of serum-corrected calcium, phosphate, eGFR, and iPTH, without calcium/phosphate metabolic diseases or renal dysfunction. Corrected calcium (cCa) was calculated by the formula: cCa (mmol/L) = tCa(mmol/L) + 0.025[40 − albumin(g/L) [14] where tCa represents total calcium.

### 2.2. Immunohistochemical Staining

The parathyroid specimens were fixed in formalin solution and embedded in paraffin blocks for routine immunohistochemical and hematoxylin-eosin staining. Paraffin-embedded 4-micrometer-thick specimens were dewaxed in turpentine and rehydrated through decreased concentrations of ethanol. Endogenous peroxidase activity was blocked by using 3% H_2_O_2_ in methanol for 15 min. The sections were incubated with trisodium citrate dihydrate liquid (0.125%, pH 6.0) for 15 min and then soaked with phosphate-buffered saline (PBS) liquid (pH 7.2–7.4) three times for 5 min. The sections were then preincubated with sheep serum for 10 min to block nonspecific antigen. The pretreated slides were incubated overnight at 4°C in a humidified chamber with antibodies to the VDR (1 : 100, cat#12550, rabbit monoclonal antibody from Cell Signaling Technology, Danvers, MA 01923, USA), CYP27B1 (1 : 100, cat#ABN182, rabbit polyclonal antibody from Upstate Technology, Lake Placid, NY, USA), CYP24A1 (1 : 100, cat#189322, goat polyclonal antibody from Abcam Inc., Cambridge, MA, USA), and PTH (ready to use, cat# MAB-0683, mouse monoclonal antibody from Maixin Biological Technology Development Co., Ltd., Fuzhou, China). After incubation with these antibodies, the slides were incubated at room temperature for 1 hour. After rinsing with PBS three times, the sections were incubated with secondary antibody anti-rabbit-HRP (KIT-9730, Maixin Biological Technology Development Co., Ltd.) or anti-goat-HRP (KIT-9719, Maixin Biological Technology Development Co., Ltd.) for 20 min, and the binding of peroxidase-conjugated secondary antibodies was detected with a DAB kit (Maixin Biological Technology Development Co., Ltd.). Hematoxylin was used for counter staining. Immunohistochemical staining of VDR in the human epidermis, CYP24A1 in the kidney, and CYP27B1 in liver was used as positive controls, respectively. PBS (pH 7.4) instead of the primary antibody was used as a negative control. Negative control slides were obtained in the corresponding tissue. Tissue for the positive control (epidermis, kidney, and liver) was preexisting and originally collected not for research purpose at the Department of Pathology of the Second Xiangya Hospital of Central South University. Use of the specimen in the present study was on the informed patient consent and approved by the Ethics Committee of the Second Xiangya Hospital of Central South University.

### 2.3. Image Analysis

The immunohistochemical staining was quantified by digital image procedures using ImageJ software (NIH, Bethesda, MD, USA) [15–20]. Tissue sections were viewed using bright-field illumination under a Leica DM LB2 upright light microscope (Leica Microsystems Wetzlar GmbH, Wetzlar, Germany). The representative areas of the different sections were captured on a Leica DFC320 digital camera (Leica Microsystems Digital Imaging, Cambridge, UK). These images had a resolution of 2088 × 1550 pixels with RGB 24 true color format and were saved in uncompressed tagged-image file format (TIFF). The same range of illumination values were used to allow maximum reproducibility to avoid differences in the illumination. Captured images were converted to gray scale in ImageJ. Cells were manually marked out with a red pencil dot in Microsoft Paint, and the dots were then automatically identified and counted using ImageJ.

### 2.4. Statistical Analysis

The statistical analysis was performed using SPSS software (SPSS Inc., Chicago, IL, USA). Data are presented as mean ± standard deviation. For the levels of VDR and CYP24A1 which comply with normal distribution, we used Pearson correlation analysis to evaluate their relationship with age. For the levels of CYP27A1 and PTH which did not comply with normal distribution, we used Spearman correlation analysis to evaluate their relationship with age. Results with *p* values < 0.05 were considered statistically significant, and all tests were two sided.

## 3. Results

**Figure 1:**
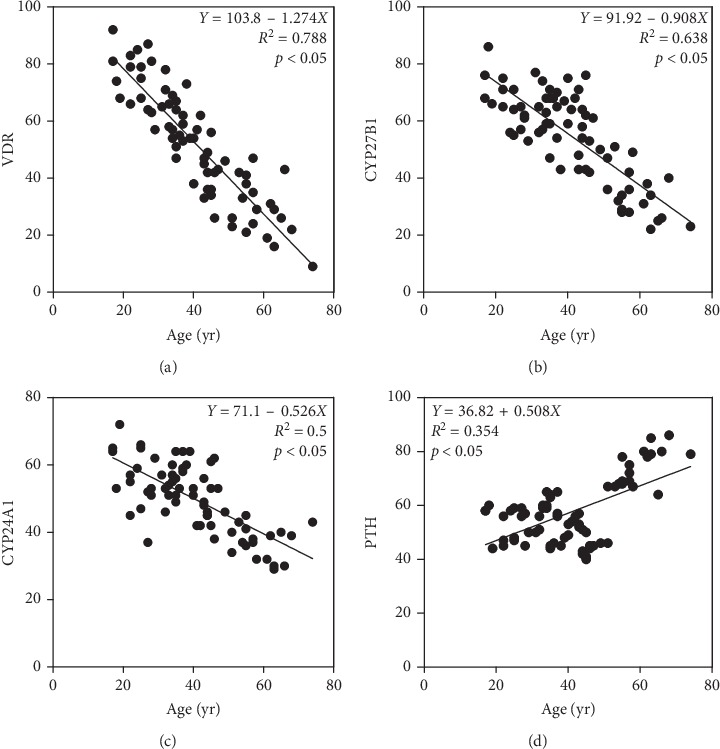
The association of (a) VDR, (b) CYP27B1, (c) CYP24A1, and (d) PTH expression levels in human parathydoid glands with age. Quantification of VDR, CYP27B1, CYP24A1, or PTH-positive cell rates in parathyroid glands of patients in different ages was performed by ImageJ software. Scatter plots of expression levels of VDR, CYP27B1, CYP24A1, or PTH versus age were shown.

**Figure 2:**
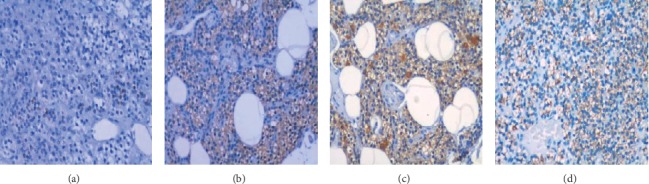
Immunohistochemical staining of (a) VDR, (b) CYP27B1, (c) CYP24A1, and (d) PTH in human parathyroid glands. The specimens from seventy parathyroid glands were fixed in formalin solution and embedded in paraffin blocks for routine histological and immunohistochemical analysis using antibodies against VDR, CYP27B1, CYP24A1, or PTH. Immunohistochemical staining of VDR, CYP27B1, CYP24A1, or PTH is shown in brown, and the counterstaining is shown in blue in the representative section. VDR is located in the nucleus. CYP27B1 and CYP24A1 are located in the cytoplasm, and PTH is located in the cytoplasm and plasma membrane. PBS instead of the primary antibody was used as a negative control. Immunohistochemical staining of VDR in the epidermis, CYP24A1 in the kidney, and CYP27B1 in liver was used as positive controls, respectively (data not shown).

To determine whether the expression of VDR, CYP27B1, CYP24A1, and PTH in human parathyroid glands is associated with age, we examined expression levels of VDR, CYP27B1, CYP24A1, or PTH-positive cells in parathyroid glands obtained from 70 patients with different ages using immunohistochemistry. Correlation studies between the expression levels of VDR, CYP27B1, CYP24A1, or PTH and age were performed. The results showed that age was inversely correlated with VDR (*r* = −0.89; *p* < 0.0001), CYP27B1(*r* = −0.74; *p* < 0.0001), or CYP24A1 (*r* = −0.71; *p* < 0.0001) positive cell rates. A significant positively correlation between PTH and age was also seen (*r* = 0.60; *p* < 0.0001). The results are summarized in Figure 1. The expression of VDR was localized in the nucleus. The expression of CYP27B1 and CYP24A1 was located in the cytoplasm, and the expression of PTH was located in the cytoplasm and plasma membrane (Figure 2).

## 4. Discussion

In the present study, we used parathyroid tissue from patients with different ages to investigate the association of expression levels of VDR, CYP27B1, CYP24A1, and PTH in parathyroid glands. These patients had nodular thyroid goiters but had no pathological conditions affecting parathyroid glands. The results indicate that a decreased expression of VDR, CYP27B1, and CYP24A1 in parathyroid glands is associated with age. This is the first report to show an association of VDR, CYP27B1, and CYP24A1 with age in human parathyroid glands.

Vitamin D has been demonstrated to regulate cell proliferation, differentiation [21], apoptosis [22], angiogenesis [23], invasion, and metastasis [24]. Vitamin D exerts most of its biological activities by binding to VDR, which belongs to the superfamily of nuclear receptors for steroid hormones and regulates gene expression by acting as a ligand-activated transcription factor [25].

VDR expression has been identified in many tissues and cell types [26], most notably monocytes and lymphocytes [27], glia [28], neurons [28], breast [29], and parathyroid [6, 7]. A number of studies have reported an association of VDR polymorphisms with Behçet's disease, diabetes, arthritis, autoimmune diseases, and hypertension [30–34]. Vitamin D levels may be affected by a number of factors including age, cultural behavior, latitude and season, and outdoor activity [35]. VDR has been found to be decreased with age in many tissues such as skeletal myocytes [9], human muscle tissue [10], rat intestine [11, 12], bone [11], and mammary glands [13]. The present data showed that VDR expression levels in human parathyroid glands decreased with age.

The synthesis and degradation of 1,25(OH)_2_D are regulated by CYP27B1 and CYP24A1, respectively. Immunocytochemical staining showed cytoplasmic staining with a microgranular pattern with antibodies against CYP27B1 and CYP24A1, which is consistent with their mitochondrial localization. We have also found a decreased expression of VDR, CYP27B1, and CYP24A1 with age in parathyroid glands. Decreased VDR expression levels in parathyroid glands in older people may lead to decreased responsiveness of parathyroid glands to 1,25(OH)_2_D, and decreased expression levels of CYP27B1 and CYP24A1 may lead to a decreased synthesis and degradation of 1,25(OH)_2_D.

The present result also showed increased levels of PTH in parathyroid glands in elderly adults compared with younger adults. These results are consistent with previous results of studies showed that iPTH levels increase with age in men and women and are about 30% higher in the elderly than in young subjects [36–39]. The increase in iPTH levels in the elderly has been attributed to declining renal function, declined calcium absorption efficiency, and declined 25(OH)D levels [3, 40]. However, other studies has been shown that iPTH levels significantly increase with age, independent of 25(OH)D levels, phosphate, and ionized calcium in the serum, and renal function [41].

Results from the present study which showed that VDR in parathyroid glands decreases with age may provide an explanation at least in part for the increase in iPTH in the elderly. Previous studies showing a decrease in the number of intestinal VDR with age leads to a decreased responsiveness of intestinal cells to 1,25(OH)_2_D have demonstrated a similar pattern for VDR in different tissue from the elderly [42, 43].

The study has some limitations. A major limitation of the study is the relatively small number of samples included in the study. Another limitation is that the assessment of VDR, CYP27B1, and CYP24A1 levels in parathyroid glands were performed by a semiquantitative method.

In conclusion, a decreased expression of VDR, CYP27B1, and CYP24A1 and an increased expression of PTH in parathyroid glands are associated with age.

## Figures and Tables

**Table 1:** Clinical features of 70 patients.

Demographic	Mean ± SD	Reference range
Age (year)	41 ± 14	
Calcium (mmol/L)	2.31 ± 0.12	2.11–2.52
Phosphorus (mmol/L)	1.04 ± 0.16	0.85–1.51
Creatinine (*μ*mol/L)	53.31 ± 5.80	44–133
PTH (pmol/L)	4.32 ± 1.36	1.60–6.90

## Data Availability

The data used to support the findings of this study are available from the corresponding author upon request.

## Abbreviations

VDR: Vitamin D receptor
1,25(OH)_2_D: 1,25-dihydroxyvitamin D_3_
PTH: Parathyroid hormone
iPTH: Intact parathyroid hormone
CYP27B1: 25-hydroxyvitamin D-1*α*-hydroxylase
CYP24A1: 25-hydroxyvitamin D-24-hydroxylase.
